# People's care seeking journey for a chronic illness in rural India: Implications for policy and practice

**DOI:** 10.1016/j.socscimed.2022.115390

**Published:** 2022-11

**Authors:** Sumit Kane, Madhura Joshi, Sapna Desai, Ajay Mahal, Barbara McPake

**Affiliations:** a Nossal Institute for Global Health, Melbourne School of Population and Global Health, The University of Melbourne, Victoria, Australia; b Gokhale Institute of Politics and Economics, Pune, India; c Population Council, New Delhi, India

**Keywords:** Care seeking journey, Healthcare seeking journey, Chronic illness, Chronic breathlessness, Chronic obstructive pulmonary diseases, Care pathways, India, Low- and middle-income countries

## Abstract

Drawing on interviews conducted in 2019–2020, across twenty villages in India, this paper unpacks how people with chronic illness navigate complex care-seeking terrain. We show how the act of seeking care involves navigating through personal, family, social, economic, cultural, and most importantly, difficult health systems spaces—and entails making difficult social, moral, and financial choices. We show how the absence of reliable and accessible points of first contact for primary care results in people running from pillar to post, taking wrong turns, and becoming disappointed, frustrated, and, sometimes, impoverished. We reveal the complex individual and social dynamics of hope and misplaced and misguided expectations, as well as social obligations and their performance that animate the act of navigating care in rural India. We shine light on how a health system with weak primary care and poor regulation amplifies the medical, social, and financial consequences of an otherwise manageable chronic illness, and how these consequences are the worst for those with the least social, network and economic capital. Crucially we highlight the problematic normalisation of the absence of reliable primary care services for chronic illness in India, in rural India specifically. We signpost implications for research, and for policy and practice in India and similar health system contexts, i.e. those with weak primary care and poor regulation of the private sector.

We argue that in India, having in place accessible, good quality, and trustworthy sources of advice and care for chronic illness at the first point of call, for all, is critical. We contend that this first point of call should be quality, public primary care services. We conclude that if such arrangements are in place in public services, people will use them.

## Highlights

• Lack of reliable, trustworthy advice and care is a big gap in India's health system.
• Being misled and suffering catastrophic consequences of ill-health is the result.
• People need to be protected from bad actors in their care-seeking journeys.
• People want reliable, trustworthy advice and care, at their first point of call.
• India's health system must urgently act to ensure appropriate care at all levels.

## 1 Introduction/background

Understanding people's healthcare and treatment-seeking has attracted scholarly attention across many disciplines. Within social psychology, the focus has been on cognitive determinants of behaviours and actions (Fishbein, 1980; Ajzen, 1991; Montaño and Kasprzyk, 2008). Sociological models tend to focus on contextual and social aspects of patients, providers, and on the relationships between health systems and other social systems; they view care-seeking as shaped by people's past experiences, and as occurring through interactions between individuals and with health and social systems (Suchman, 1965; Igun, 1979). Understandings rooted in an anthropological perspective tend to explain people's care-seeking based on their beliefs about origins and causes of illnesses, and through an understanding of symbolic meanings, motives, feelings, and stresses that people experience, associate with, or assign, to the illness, ill-selves, and illness experiences (Hahn and Kleinman, 1983; Rhodes, 1996). The critical social sciences perspective argues that the act of seeking care is socially produced at the intersection of economic status, class, gender, and societal norms and relations. It contends that health-care decisions are best examined as being influenced by individual, social, and cultural constructions (identity, beliefs, values, relational dynamics in family and society) as well as wider socio-political-economic forces (Singer, 1989; Lupton, 1995).

While these conceptual works largely (Igun being the exception) draw from experiences in high-income contexts, they have widely informed empirical research on care-seeking in low-and middle-income countries (LMICs), including India. In India, curative health services, particularly primary care services, are predominantly (approximately 75%) provided by private, for-profit healthcare providers. These providers range from those who hold formal qualifications in Allopathy, Ayurveda, or Homeopathy, to a whole range of informal, unqualified providers; qualified healthcare providers and services (particularly Allopathic/Modern medicine) are concentrated in urban areas (Das et al., 2020; Kasthuri, 2018). The health system is poorly coordinated and poorly regulated, with few quality assurance mechanisms – for instance, the dispensing of modern medicines by unqualified and inappropriately qualified providers is common (Mohan et al., 2008; Calnan and Kane, 2018; Gopal, 2019). Research on care-seeking from India includes studies describing and explaining under-utilisation of formal health services and/or the use of traditional healers, unqualified providers. People's actions are explained as underpinned by factors such as traditional belief systems (Mohan et al., 2008; Chomat et al., 2009), lack of confidence in formal services (Mohan et al., 2008; Bhanderi and Kannan, 2010), financial constraints (Chomat et al., 2009; Bhanderi and Kannan, 2010), stigma and taboos (Bhanderi and Kannan, 2010; Agarwal et al., 2007). These studies are from across India and relate to different illnesses. Some also discuss what has been described as ‘patient navigation pathways’ (Selvam et al., 2007; Pati et al., 2013; Kapoor et al., 2012) – referring to various services used, sometimes providing the rationale for choosing a course of action, or for discontinuing use of one provider and moving to another. We build on this literature, and in this paper, reimaigine care-seeking for chronic illnesses in contexts such as India's as a journey involving deft social navigation (Vigh, 2009).

We argue that the act of care-seeking is significantly different in LMIC contexts as health systems tend to have weak primary care, poorly functioning referral arrangements, and weak regulation and oversight of often a large and diverse private healthcare sector (features similar to the Indian health system). We contend that expanding the ways in which we understand care-seeking in such contexts can yield valuable insights for policy and practice. In this paper, through examining care-seeking for a chronic illness (chronic severe breathlessness as a tracer condition we unpack how people experience and navigate care in rural India. We draw attention to the complex individual and social dynamics of hope, uncertainty, and expectations that animate the act of seeking care. We highlight the significant, albeit problematic, role of the health system. In doing so we signpost implications for health policy and practice in India and other similar health system contexts.

## 2 Methods

Findings presented here are from a multi-methods study that explored healthcare seeking for a chronic illness in two different health system and development contexts (states) within India - Uttar Pradesh (UP) and Maharashtra (MH). The former is ranked at the bottom, while the latter sits at the top end of the national health system performance index (NITI Aayog, 2019). This state level index is a composite of 24 indicators across the domains of Health Outcomes, Governance and Information, and Key Inputs/Processes. The overall study aims to understand people's care-seeking actions, choice of providers, costs incurred, and care-seeking journeys of those experiencing chronic breathlessness at rest or on minimal effort, for more than 6 months (inclusion criteria for the broader study). The broader study involves three components - household survey, discrete choice experiments, and a qualitative inquiry. This paper reports findings from the qualitative inquiry conducted in 20 villages across Uttar Pradesh and Maharashtra states over a period of 8 months in 2019–2020.

As part of the qualitative inquiry, we conducted in-depth interviews with 41 purposively selected respondents (meeting the inclusion criteria for the broader study). Participants were recruited through a three-step process. In the first step, in the selected village all those households with a member with the condition were identified for inclusion in the sampling frame for the broader study (the line list); a preliminary consent for inclusion in the broader study was sought; basic demographic and household characteristics were recorded. Identification was done with help from the local community health worker and village elders. Step 2 - at the end of each day, the study team discussed the line list to identify those most suitable for inclusion in the qualitative inquiry (criteria: likelihood of being insight rich, based on the initial interaction). Step 3 - those identified were approached for an initial interaction on the next day; and from amongst these, those judged most suitable for inclusion in the qualitative inquiry (as assessed by the 1st and 2nd authors) were interviewed.

Of the interviewees, 22 were women (UP-13; MH-9) and 19 were men (UP-11, MH-8); ages ranged between 35 and 80 for women and 45 to 82 for men. The vast majority of participants identified themselves as being of modest means or poor. All interviews were conducted in participants’ homes and were often conducted with more than one person present and talking. Our requests for textbook privacy during interviews were often met with the following kinds of responses from participants: *“These people can stay”* or *“There are no secrets in our family”*. This is consistent with our experience of conducting research in rural India. In our analysis we account for the presence of others during the interview. All participants provided written informed consent; interviews were audio recorded and transcribed verbatim in the interview language, translated to English (all transcriptions and translations were checked by one of the co-authors; random checks were made by the first author), and coded/analysed using NVivo; all transcripts were anonymised to remove personal identifiers and location details. Ethics approval for the study was granted by independent ethics committees at the National Council For Applied Economic Research, New Delhi, and The University Of Melbourne, Australia.

### 2.1 Analysis

Guided by Atkinson (2017), and Radley and Billig (1996), in our analysis we treat people's accounts as “expressive and constitutive”, as “accounts of a disclosing of a supposed internal attitude … (and) involves situated claims and justifications”. We consider the narratives proffered by participants as performative “speech acts” and expressions of “socially shared resources” (Atkinson, 2017 p 5). This could involve the use of rhetorical devices to socially represent oneself, or one's position with respect to others or the social norms, or performative elements enacted in the presence of others, and the use and invocation of “discursive resources and conventions” (Atkinson, 2017 p 6) to produce private, public or a combination of these accounts. Analysis was iterative and involved repeated readings of interview transcripts and sometimes listening of interview recordings. Analysis of form and function was given particular importance to explicitly uncover nuances that would be overlooked or missed if verbal accounts were treated with a thematic analysis approach alone. Memos and annotations were developed, expanded, and refined throughout - during fieldwork, analysis, discussions between co-authors, and while writing the manuscript.

Our analysis is informed by Vigh's (2009) work which makes the case for applying the concept of navigation when studying people's interactions with the social world, as it allows one to better analyse the “way people not just act in but interact with their social environment and adjust their lives to the constant influence (in potentia and presentia) of social forces and change” (p433). Vigh contends that this is appropriate as navigation entails the act of “moving across a moving environment” (p428) and it “emphasizes the construction of tentative mappings and a constant dialogue between changing plots, possibilities and practice” (p429). To us thus, imagining care-seeking as an act of social navigation is very apt as social navigation “encompasses both the assessment of the dangers and possibilities of one's present position as well as the process of plotting and attempting to actualize routes into an uncertain and changeable future” (p425).

## 3 Findings

We present our findings and analysis along two intertwined tracks. We recount the steps and the experiences of a typical care-seeking journey; concomitantly, we reveal how study participants, together with their significant others, navigate the journey; throughout we examine what drives and underpins people's manoeuvres. The findings typify the experience of those of limited financial means – the vast majority of our study participants; the experiences of those with better means are used to reflect on these experiences.

### 3.1 The beginning: running from pillar to post

While the beginnings of the illness were often blurry for most, the start of the care-seeking journey always seemed to be a vivid memory, often described the start of their journeys with reference to and linked to an acute, major exacerbation of the illness and marked by a flurry of activity – often rooted in a desperate search for answers and for a ‘cure’. A universal sentiment at this stage was articulated by an old woman who said that she visited *“whichever doctor was available nearby … we may have gone to at least 10–15 doctors”*, emphasising that she *“wanted to be cured no matter if it was at a government hospital or private hospital” (Elderly Woman-UP).* We found people desperately consulting with and seeking care from a wide range of providers at this stage of their care-seeking journey – often in parallel. That said, we recognise that both experience/observation and research on care seeking points to some kind of ‘internal’ care decision making personally or within the household when they someone first starts not feeling quite themselves. This includes ‘let's watch a bit and see’ – to do nothing for a while is still a decision. While respondents wanted to talk about the acute, major exacerbation of the illness as the start of their care-seeking journey – the blurriness (about the absolute beginnings) we encountered in most respondents most likely includes this initial ‘internal’ decision making process.

While some people first tried home remedies and traditional medicines (especially in UP), most turned to one of the many unqualified providers dispensing Allopathic medicines nearby, the so called ‘Jholachap’ providers. In the thirty-odd villages we visited over the course of our fieldwork, all had at least one, often multiple such unqualified providers. These providers typically charged a modest fee, and dispensed Allopathic medicines and injections; they were by far the most common port of first call for study participants. The term ‘Jholachap’ literally means someone who has nothing but a bagful of medicines to show for themselves; the term has a dismissive, slightly derisory connotation to it. In referring to these providers as ‘Jholachap’, our participants were unequivocally communicating the clarity of their understanding about the status of these providers, and in yet using their services, suggesting either that they were doing so with full knowledge of what they were doing or had no choice but to use their services. However, when queried, while generally all could identify a ‘Jholachap’, many could not explain when a provider was formally qualified. The typical response to queries regarding provider qualification was “we know” – suggests some sort of a collective, socially constructed understanding. Another port of call were qualified providers (with qualifications in Allopathic medicine ie., MBBS/MD), typically further away from home, at either the sub-district or district level. People consulted these providers in both private and public settings, including occasionally at the public primary health centres in, or close to, their villages. Lest this account suggest an orderly progression from one type of provider to the other, in the early stages of illness the reality with rare exceptions was that people were zigzagging between and trying out many different providers, sometimes simultaneously.

The hope for a cure, and of getting rid of the illness by its roots, as encapsulated by these oft repeated phrases *‘Jad say nikal jaye’* in Hindi, and *‘Mulaatna nighoon jaava’* in Marathi (translation: It should go from its roots), underpinned and overwhelmingly shaped people's actions at the start of their care-seeking journeys. The notion of diseases having roots, is built around the metaphor of the disease as a weed infesting the human body and mind, and the idea that a true cure involves the uprooting and weeding out the disease. This notion is common across India, and one finds similar phrases across many Indian languages. These hopes for a cure drove people to run helter-skelter between the Jholachaps, providers of traditional medicines, qualified providers of Allopathic medicine, and between private and public settings – searching for answers, and as articulated by one of the study participants, desperately seeking a cure “*Yes. I wanted to be cured … no matter what!*” *(Elderly Woman – UP).* The quotes presented in this section exemplify what shapes people's care-seeking in the early stages of any major illness in the study context. This mix of hope and desperation for a cure drives people from pillar to post, often in circles, and as we discuss further later, drives the vulnerability to being financially exploited by unscrupulous providers.

### 3.2 Leaving no stone unturned

While the quest for answers and the hope (and desperation) for cure were the driving force behind people's care-seeking decisions and actions, they were underpinned by social (cultural, relational, and moral) dynamics. Without exception, the process of seeking care was a family matter. No matter if the person affected was the main breadwinner (often a man) or the main homemaker (often a woman) or an older member of the family (typically a parent), the immediate family, and often also the close relatives, articulated a unified narrative to us - that of standing in solidarity with each other. In the interviews, across contexts, study participants vociferously referred to the support they had received from their relatives. We realised that this consistent referral to their relatives' solidarity was clearly meant to perform multiple moral, relational, and social functions, particularly given that interviews often could not be conducted in absolute privacy. The references served as public acknowledgment and expressions of gratitude; as affirmations of the social norm whereby one was expected to stand by one's relatives and to be seen as someone who did so; and also, a reiteration and a reminder of the continued existence of these moral social expectations. The overall narrative, of mobilising all possible resources to find a cure, and of solidarity while doing so, was also meant to communicate, to self, to the patient, to relatives, to society at large, and to the interviewer, that all that could have been done to find a ‘cure’ had been done, and that no stone had been left unturned.

In as much as care-seeking was underpinned by social dynamics, we also found that people's decisions and actions - the proverbial stones turned - were circumscribed by hard-nosed, real-world, intra-household financial considerations and calculi. Decisions ultimately came down to how much money the family had and could spare for treatment. During interviews there was palpable tension between the narrative of leaving no stones unturned and the intrahousehold allocation of resources, and the contingent care-seeking decisions and actions. Interviews exposed the centrality of money, and how it ultimately trumped other social dynamics for those with little means. Those who were poor brought up money very early on in the interviews and repeated it several times. Those with better means typically raised the point later in the interviews; and if the interview was happening in a not so private setting, they made sure that they made the point obliquely through gestures, often discreetly. Those few who were well off never brought up the question of money – when however explicitly asked, they laughed it off, often offhandedly dismissing the amounts spent as too trivial for them to care. The centrality of money, and the near fatalistic approach to one's possibilities therein, is illustrated by the following excerpts – as is the absence of accessible, affordable, good quality care.

“Poor people are left with no other option but to go to the local (unqualified) providers … irrespective of the treatment they receive. What else can they do? People who have money can afford going to big cities like Delhi, Kolkata, Mumbai. The poor can only go to local doctors and leave the rest to God.” (Man, 82-Yrs, UP)

The notion of leaving no stone unturned for one's loved ones, in its various facets, is, our findings suggest, at the heart of what underpins much of what has been called ‘distress health financing’ and is also a harbinger of experiencing ‘catastrophic expenses’. Our findings also suggest that the moral, relational, and social symbolic importance of this notion in the study communities was very high. The public proclamations (in front of us, and also generally) of the act of leaving no stone unturned for one's loved ones, expose both, the failings of the health system, not least the lack of reliable and good quality primary care services, as well as the lack of health-related risk protection in the study communities.

### 3.3 Wrong turns and blind alleys

In the quest for answers, and in the personal, relational, social, and moral imperative to leave no stone unturned, and in the desire to communicate to society that no efforts had been spared in the search for ‘curing’ what was essentially a chronic lifelong (incurable) illness, study participants and their family members took many wrong turns and stumbled into blind alleys. While the nature of people's paths varied, a key common feature across journeys was the lack of reliable information and guidance about the illness and about appropriate treatment – critically, at the beginning of the care-seeking journey, but also beyond. This meant that people had to rely on their social networks to make sense of their illness and to chart a response. Who people turned to varied widely, but the difference between those with high social capital and others was however merely in the degree and extent to which they ran into blind alleys and bore the expense of uncertain turns; the only exception being those few who had some qualified healthcare providers in their social network, and those few who by chance happened to find the right starting point in their care-seeking journey. Again expectedly, those with high social capital also happened to be those who could afford some missteps, and those with low social and network capital were typically poor and could not afford even the smallest of missteps.

The starting point stood out as the pivotal moment in people's care-seeking journeys; we found that it disproportionately determined the paths people took. It set the stage for: vulnerability to being misled, to receiving inappropriate care, and to being exploited. Of the twenty villages we conducted interviews in (ten each in UP and Maharashtra), only four villages had one or more qualified (MBBS) doctors' private practices; these four bigger villages also had public primary health centres which had one or more qualified (MBBS) doctors in place. All villages, including the big villages had unqualified providers, the aforementioned ‘Jholachaps’ peddling their wares. The following excerpts highlight how the encounters with these unqualified providers set people on particular care paths.

“Actually, what happens is … here there are a lot of unqualified doctors [ …]. If you are taking their treatment and if you don’t get better, they will just send you on to a particular doctor. Their main motive is to get a commission”.

“Even if there is a minor ailment, these private doctors (unqualified and qualified alike) will exaggerate it, and say it is serious, and recommend that you go to […] big cities but won’t recommend going to government hospitals.” (Man,56-Years, UP)

Laced with varying degrees of animation and with emotions ranging from anger to indifference, some versions of these accounts were proffered by almost all participants. Without exception, people presented these accounts as a given and enunciated them with absolute certainty; there was some anger, but rarely any outrage that would suggest an expectation of something better. Those who were thus set-up to walk the long and unknown path, the vast majority of our respondents and their families, expectedly experienced many difficulties in this early stage of their care-seeking journey. These ranged from not getting answers to questions about their illness and treatment options, being misinformed, being inconvenienced, not getting relief, continued suffering, receiving wrong and improperly administered treatments, suffering from serious side-effects, and spending far more money than necessary.

### 3.4 Disappointment, frustration, and penury for some

This disease has broken me in all ways possible. (Man – 59-Years, UP).

This search for cure from its ‘roots’, the social imperatives to leave no stone unturned, to be seen as having done so, and in the process encountering many blind alleys and wrong turns, was a disappointing and frustrating experience for the vast majority of our study participants. It was also an expensive affair; particularly if the patient happened to be the main breadwinner of the family. While the above quote refers to ‘me’, the disappointments and frustrations took many forms and applied as much to the immediate family. We highlight this in the next paragraph through presenting a first-person account of one of the authors' interactions with a middle-aged man from Maharashtra, and his adult daughter.

When I asked him how he felt about his illness and the expenses incurred, his daughter interjected animatedly to say, *“He feels really bad about it, what else will he feel?“.* With tears in her eyes, she added *“He feels like ‘I have become a burden on others’”*. I acknowledged her feelings and her response, and again turned to look at him, making eye contact to urge him to share his thoughts. He stayed quiet and lowered his head; now with tears flowing down her cheeks, the daughter interjected again *“He does feel like that. I can feel it before he can say it. I think he feels that ‘in our old age we have become a burden on our daughter’“.* He looked up at me with tears in his eyes as his daughter reached out to touch his arm in silence; breaking the silence, the daughter added with pride in her voice and demeanour that her father was an honourable man, who, till this illness struck, could easily climb and harvest 150 date-palm trees every day.

Patients and family members across the board, echoed this despondence, often articulating their experience, like this middle-aged man did, using the metaphor of a battle *“The disease has overpowered me … I have been defeated at the hands of this disease” (Middle-Aged Man, MH)*. This sense of disappointment with oneself (as individuals and as a family) and the framing of the experience as personal failure and defeat *“My strength is no match … I have succumbed to the power of this illness (‘bali gelo’)”,* was similarly a recurring theme. The phrase “*bali gelo”* in the Marathi language articulates a sense of dignified despair and defeat (often in a battleground setting). The phrase captures the essence of the experience of many a study participant - particularly those who were poor or were breadwinners in the family. Further, as the following quote illustrates, people saw their situation as a personal responsibility and failure “*I would have done all the work, I would have toiled, I would have continued and not quit my job […] … my … our … strength has fallen short” (Man-62 Yrs, MH).* This discourse somehow also reflects the low expectations people have of the health system; it also highlights the absence of any meaningful mechanisms to protect people from health-related risks.

The various ways in which illness, particularly chronic illness and its care can have distressing financial consequences for individuals and families is well-documented in India (Rajpal et al., 2018; Kastor and Mohanty, 2018). Consistent with this literature, as excerpts in Box 1 illustrate, participants in our study reported resorting to selling assets, incurring debt, reducing essential expenditures, and foregoing opportunities e.g., children's education. Not unexpectedly, families where the main breadwinner was the patient, were the worst off, and were the ones likely to be driven to penury. The invocations of resorting to begging that one sees in the quotes in Box 1 were both literal and metaphorical. Culturally and linguistically, such talk of ‘begging’ connotes the lowest possible point one could fall to socially. This talk was common amongst those study participants who were poor, and the talk was always laced with a mix of frustration and despair. However, except the few who had been totally “broken” and driven to penury by the illness, these invocations almost always also had a defiant tone – a defiance of the power of the illness and of the circumstances it had led one to.

Box 1 We had to sell all of the three plots (of land) that we had … all that money was spent on his treatment. (Son-UP) If our father would have been fine, then we would have continued our studies … we had to leave our studies. (Daughter-UP) We literally begged […] we borrowed money from our relatives and friends! (Elderly couple-MH) If we don't have money, then we will beg, we will get a loan […] we will accept whatever they have to offer. (Family-MH).

Alt-text: Box 1

### 3.5 Learning, accepting, and living with the illness

The disappointments, frustrations, and financial distress notwithstanding, as time went by, all participants, each in their own unique way, had come to understand the nature of their illness and come to terms with their situation. While people continued to talk about the proverbial ‘cure from the roots’, they were no longer actively searching for it. They had come to realise what one of the participants so poetically said *“Yeh dam tow dam kay saath he jaayega” (Man-UP).* In Hindi and Marathi alike, the word ‘dam’ is used to refer to both, being breathless and to being alive, and what he was saying was that he now recognised that his illness would only go with the stopping of his breath (dying). Unlike the defeated acceptance of the early stages of illness and seeking treatment, this acceptance was that of a battle-hardened soldier knowledgeable about the enemy and the enemy's powers, and confident about one's way about the illness and its treatment.

This knowledgeability was akin to the ‘expert patient’ concept within the literature on chronic illnesses (Donaldson, 2003). Study participants recognised the waxing and waning nature of their illness; knew what would trigger a flare-up; knew what could help prevent flare-ups; what treatment worked for them, and where it could be had. The expertise of the participants in our study however extended further. Each had developed a triage algorithm of sorts of their own, with personalised criteria for when to go for a certain kind and level of treatment, and where/whom to go to or not to go to. Unlike much of the literature, in our study this ‘expertise’ resided not in the patient alone, but rather within the immediate family as well. This collectively held expertise was also unique in that it was simultaneously certain and tentative; the former related to having learnt lessons the hard way, and the latter because it was constantly debated, contested, and negotiated – primarily between family members, but also with others in the social network and with providers as well as with constraints in available care and finances. This knowledgeability and acceptance were also related to patients and family members coming to terms with the chronic life-long trajectory of the illness and treatment, accepting a revised identity of oneself, within their immediate family, within their social network, and sometimes, jointly crafting a new identity.

## 4 Discussion and conclusions

Through our findings we have illustrated that seeking treatment for a chronic illness in the context of a weak health system like that of India's, is demanding. It requires active navigation to traverse across a complex and varied terrain of overlapping personal, family, social, economic, cultural, and most importantly, difficult health systems spaces. The notion of a journey without a clear path, many uncertain turns, and blind alleys, and of navigation across uncharted terrains is particularly apt given that people do not know what they are suffering from, have no way to know the right places to seek advice and treatment from, have few ways to know which provider is appropriate and trustworthy, and have no reliable mechanisms to understand what outcomes to realistically expect. Foregrounded by the literature, and referencing these findings and reflections, in this section we reimagine and articulate care-seeking for chronic illnesses in such contexts as a two-stage journey, with each stage consisting of multiple steps, and the act of care-seeking, as an act of social navigation (Vigh, 2009). Stage 1 being about Urgency, Search for cure, and Hope, and, Stage 2 about Reconciliation, and Acceptance. We discuss the nature and salience of such an understanding in the context of health systems with poor primary care services and reflect on the implications for care for chronic illnesses in such contexts.

### 4.1 Stage I: urgency, search for cure, and hope

In contexts where the health system is not organised to provide appropriate advice and care, a new illness that affects someone's life and livelihood expectedly triggers a flurry of actions. And given that choices and possibilities are only constrained by financial resources and will (personal and social) - what appears to be a helter-skelter response involving turning to all kinds of providers, is in fact the only possible path for many. This initial stage of the care-seeking journey expectedly involves making sense of the problem and is marked by and driven by ‘hope’ - the hope that the illness might be cured.

In this stage, people seek out those who they think might ‘cure’ them – and given the context, run from pillar to post to try the wares offered by a range of services and providers. This search for ‘cure’ and ‘cure from the roots’, intuitive as it might seem, is problematic at many levels. This expectation of getting ‘cured’ and the common cultural notion that all illnesses have roots and if these roots are weeded out, there will be a permanent cure, is what drives people to run from pillar to post. While in many acute and often infectious conditions this metaphor applies - in almost all chronic conditions like the chronic breathlessness in our study, this expectation is misplaced. This notion was so deep rooted amongst our study participants that despite being told that the problem could only be controlled, and not ‘uprooted’ and cured, many continued to search for the proverbial cure till they had exhausted their means. Finally, our findings demonstrate how it is the absence of responsive, reliable, and easily accessible providers who would direct people in the right direction, both in terms of appropriate expectations and appropriate care, that drives people down the path of desperation, disappointment, frustration, and sometimes penury. In this stage, given this absence of reliable advice and care, the inconvenience experienced is accepted as the norm, even worn as a badge of honour.

We therefore contend that this first stage, characterised by worry, an urgent search for cure, and hope for cure is the time when people are most desperate and therefore most vulnerable. Our analysis suggests that only the few who happened to receive appropriate advice at this stage were spared the running from pillar to post and the expenses that follow. For almost all, in this stage, the desperation when compounded by the lack of reliable and trustworthy sources of advice and care, renders people vulnerable to both, being misled down wrong paths, or/and being exploited by unscrupulous, profiteering providers. Vulnerability is amplified at the intersection of worry, the urgent search for and hope for cure, the desire to do the right thing for one's loved ones, and the desire to be seen as doing all that is possible for one's loved ones. We found that this convergence manifests itself in the form of people resorting to impoverishing distress spending in the form of sale of assets, borrowing at extortionist rates, and foregoing of educational opportunities for family members.

Having said this, it important to note that across the board study participants seemed to look back to this stage of their care-seeking journeys with a mix of suffering, yet satisfaction and pride. The typical narrative turns involved suggestions to the effect that ‘we did all that could be done’, ‘we left no stone unturned’, ‘we went into debt’, ‘we did the right thing even while incurring great expenses and despite it entailing all kinds of sacrifices' while at the same time lamenting the suffering and stress experienced during that stage. To us this narrative of suffering and pride in the sacrifices made and agency exercised, was multi-layered. We as interviewers realised that there were three key audiences for this narrative – the interviewer, the immediate family who were involved in caregiving, and the community where the family resided; each party approached the narrative from a different angle. The interviewer was an incidental audience - we felt that respondents were using the interview process as a platform to express their feelings. At one level the narrative was an acknowledgment by the patients of their gratitude towards those who provide for and care for them. At another level, the narrative served as a not-so-subtle reminder of one's social duties to those who were meant to be responsible for caring. For the provider-carers the narrative was a way to reassure themselves that they had fulfilled their responsibilities. It was also a way for the carers to reiterate and reassure the patient that they remained committed, and a way to communicate to the community that they were and will continue to fulfil their socially prescribed and expected responsibilities. Crucially, from a policy perspective, this narrative poignantly reveals the very problematic normalisation of the absence of reliable primary care services in India, in rural India specifically.

### 4.2 Stage II: reconciliation, acceptance

By now the patient and his/her family have gone down long-winded paths and tried a wide range of providers. They have heard different point of views, have often undergone different diagnostic tests; their curiosity has been whetted or sometimes exhausted, and they have arrived at some understandings for themselves. They have settled on a name for their illness; it is not necessarily a technical diagnosis but rather a social understanding that often includes a well-known label (in the case of chronic breathlessness – it is ‘Dama’). They have arrived at a causal narrative too. By the time they get to this stage, most patients have also negotiated (and accepted) a revision of their identity - with themselves, within their immediate family, and within their social network.

All respondents and their family members had arrived at some form of acceptance of the chronic and lifelong nature of the problem and had a sense of reconciliation of living with the illness, and to the idea that there might be no cure. This stage is marked by a kind of knowledgeability and certainty –a confident embodiment of the sick role. All patients by now knew for certain what triggered exacerbations and worsened their condition; they were certain in their knowledge and pushed back against any suggestions that challenged this knowledge. All patients by now knew for certain what medicines provided relief; without exception, study participants decided and took these medicines for themselves irrespective of whether the medicines were technically correct or not, and irrespective of whether they were appropriately monitored for side effects. Amongst those at this stage we observed several instances of the inappropriate use of steroids - this became clear from the medicines being used (all interviewees wanted to show their medicines), and sometimes it was clear from the presence of signs of steroid over-use (one of the authors is a medical doctor). While the outcomes across types of providers were not ascertained, we observed that individuals treated consistently by trained providers, particularly by qualified Allopathic doctors, were on correct treatments, and were spending less on medicines.

In this stage, the ‘experienced-expert patient’ knew for certain which provider they would turn to and when – they were no longer running from pillar to post. Their choices were strategic, often private, always calculative, and pragmatic, but not always medically ‘right’, or safe. Patients and their family members were certain about who their providers were (often people had a few preferred providers) for day-to-day care, and for episodes of exacerbation. People's expectations in this stage were no longer about getting ‘cured’ but rather about getting ‘relief’; these changes in expectations are reflected in the preferences and choices of providers – convenience and responsiveness become critical in this stage. Thus, this second stage is characterised by exhaustion of one's possibilities and resources, by reconciliation and acceptance of one's situation, and by emergence of health service users (patient/family) who are calculative, pragmatic, and focused on getting relief, on convenience, and on the responsiveness of providers - such a sharp focus however creates its own unique risks. In this stage, patients, and their families need reliable, good quality, and responsive sources of advice and care, close to their homes.

### 4.3 A novel approach to understand care-seeking for chronic illnesses in contexts like India's

Examining care-seeking as a journey to be navigated is not totally new. Our study participants' unique experiences of navigating the various spaces and relational webs, resonates with the concepts of “healthcare pathways” or “therapeutic itineraries” or “patient navigation pathway” in the literature (Selvam et al., 2007; Pati et al., 2013; Kapoor et al., 2012). However, this two-staged care-seeking journey, with each stage consisting of multiple steps requiring deft social navigation across an uncertain terrain (i.e contexts where health systems are weak, poorly regulated, and unreliable), builds on Vigh's (2009) work to offer an original representation of the act of care-seeking for chronic lifelong illnesses in such contexts. Our paper offers both, a novel explanatory heuristic, and an example, to understand and unpack care-seeking in such contexts.

We demonstrate that the act of seeking care involves navigating through complex personal, family, social, economic, cultural, and most importantly health systems spaces, and that it entails making difficult social, moral, and financial choices. While people overwhelmingly talk of financial constraints as underpinning and driving their care-seeking, our analysis shows that a financial explanatory program alone is insufficient to explain care-seeking, particularly for chronic long-term illnesses. The imperative to leave ‘no stone unturned’ is in many ways the key driver, and the financial and other practical constraints are the only brakes to that driver. While we acknowledge the relevance of financial considerations, we show that care-seeking in fact involves deft and calculative social manoeuvrings and navigation. We highlight that the effectiveness of this navigation is determined by the cultural, relational, and financial resources at one's disposal, and, by where one is in one's care-seeking journey (Vigh, 2009) – for a significant subset of the population, usually the poor, this translates into never being able to get beyond locally available care, in the study context, provided by unqualified ‘Jholachaps’.

We show how, for the act of seeking healthcare, the locus of control, the driving force for action and exercise of agency in matters of care-seeking, may lie not only, nor necessarily in the individual, but also within surrounding structures and relations which serve as the source of an individual's advantage or disadvantage. That the act of seeking care is best understood broadly - as being constantly shaped by a person's, a family's, or a community's identity and norms which themselves are constantly evolving. While others have reported similar findings on care-seeking in contexts with weak health systems, our analysis is novel in that we emphatically show that in such contexts, responding to illness and the act of care-seeking involves multiple steps, often in different directions, and that the care-seeking journey entails a much more dynamic, reflexive, and negotiated process of navigating the social and cultural, health system, and economic maze. The other major policy and practice insight we offer relates to the role played by ‘bad’ starting points (unqualified and qualified providers alike) in people's care-seeking journeys. These early interactions are critical – if they do not go well (even to a small degree) they are potentially catastrophic as their overall impact is to exacerbate the many weaknesses of the health system rather than to mitigate them.

### 4.4 A failing health system, and implications of these failings

What our findings really tell us is a tale of the health system failing to protect citizens in the time of their need, reflecting a lack of appropriate services as well as risk protection. That study participants and their loved ones had to take on full responsibility for making complex health and care related decisions because of the absence of a reliable primary care system, stood out as a key finding. This has long been the case, but epidemiological transition, which has been dramatic in India over the last 25 years (ISLDBIC - India State-Level Disease Burden Initiative Collaborators, 2017) greatly exacerbates these problems. While acute illness has always played a role in maintaining households in poverty, the potential for it to ultimately be resolved implies that the financial risk is finite. Chronic illness never stops draining households of their resources, and the futile, helter-skelter, initial search for a cure proved capable of impoverishing even those households whose narratives appeared to start in positions of relative economic security. In India, the prevalence of Ischaemic Heart Disease has been estimated as approximately 14% in 2013 (Prabhakaran et al., 2016); prevalence of high blood sugar is above 10% in most states (ISLDBIC - India State-Level Disease Burden Initiative Collaborators, 2017); and prevalence of Chronic Obstructive Pulmonary Disease is estimated at approximately 7% (McKay et al., 2012). In other words, most households will need to navigate these health system hurdles at some point. Further, the Indian population tends to develop cardiovascular disease at younger ages, with the average age of a myocardial infarction being 52 for South Asian populations compared to 62 for those of European origin. This implies that it will often affect major breadwinners in households, whose illness has the greatest economic impact on households. The potential for chronic illnesses to be a major economic development and social problem for India is clear.

The critical health system gap is the absence of reliable primary care that is physically and economically accessible to people. Other literature from India documents widespread issues with public health services at all levels – ranging from poor technical quality, poor amenities, to providers being rude, insulting and/or humiliating (Kasthuri, 2018; Gopal, 2019). However, far more common for our respondents was the unavailability of a reliable source of first-line care – either due to physical distance or cost (e.g Das et al., 2020) or sometimes, lack of trust in public services. Respondents' accounts indicate that the choice of first provider is critical. It can support a faster and less expensive realisation that the problem cannot be cured and an earlier transition to the less economically damaging steady state where the symptoms of the condition are managed. The absence of good quality, affordable and close to community health care provision also explains the presence of the multiple informal, at least superficially affordable providers, although their low price misrepresents the potentially catastrophic economic and health consequences. These providers include the ‘Jholachaps’ whose character appears well understood by the community but who still find many customers among those who find themselves lacking alternative options. They also include a wide range of providers whose characteristics are not easy for community members to discern; who do a better job of imitating qualified providers; or those who may be qualified but act in bad faith for profit at the expense of their clients' health and economic well-being. All these providers rarely make a positive contribution to the navigation of the health system by our respondents and much more frequently cost them scarce financial resources for no return and send them to further providers who may do so on an even larger scale.

A final point that came as a surprise to the research team. We had selected the two states with a view to understand the extent to which a state with better health outcomes and a stronger public health system (Maharashtra) would have qualitatively better health system navigation patterns relative to one in which health outcomes and the public health system are weaker (Uttar Pradesh). In fact, the care-seeking journeys and accounts were remarkably similar. Although the qualitative inquiry was not set up to explain differences in experiences between the two health system contexts, our findings suggest that while reliable public health care providers may well be more prevalent in Maharashtra than in Uttar Pradesh as is commonly believed, they were not in the reach of the categories of households we interviewed or were not geared to treat chronic breathlessness, perhaps both. It is possible that the lack of trained providers in general, and higher costs associated with chronic illness in particular, resulted in similarly (difficult) navigation pathways in both states. Das et al. (2020) have also reported a high proportion of untrained providers at primary care level in both states. Also, we have not conducted research on the supply side in the two States, so offer these inferences somewhat tentatively, along with the important caveat that we could not and did not assess or compare the quality of care provided or health outcomes in this study.

### 4.5 Implications for health policy and practice in India

We have used Chronic Breathlessness as a tracer condition to understand people's experience of seeking care for chronic illnesses in rural areas, and while there might be variations across illnesses, the chances are that experiences are similar given the state of health system across the two study sites and India broadly. Our findings translate into two interrelated sets of implications for health policy and practice in India, as well as for similar contexts where primary care services are weak and health systems are not organised to address chronic illness.

- The rising burden of chronic diseases amplifies the longstanding need for good quality, reliable, and easily accessible healthcare services in rural India. It adds urgency to the need to protect the health and well-being of individuals as well as to protect their wider family from impoverishing long and winding journeys to appropriate care.
- To protect people from being misled and experiencing catastrophic consequences of ill-health, one needs to intervene differently in different stages of illness, and crucially, people need to be protected from bad actors at all stages of their care-seeking journey.
- Having in place accessible, reliable, and trustworthy sources of advice and care at the first point of call, for all, is critical. If and when such arrangements are in place in public services, people will use them. While the challenges involved in making this happen are well-documented (e.g., Das et al., 2020), recent work by Mulcahy et al., 2021 demonstrates that modest investments can yield significant gains.
- Direct regulation of the private health sector in India is a Herculean task (Calnan and Kane, 2018; Das et al., 2020) – however it needs to be done. For primary care in rural areas of India, indirect regulation by competition (Mc Pake and Hanson, 2016) – i.e., strengthening and improving public services may be a feasible route to subvert bad actors (unqualified and qualified alike) and to crowd them out of the healthcare market.

## Credit author statement

Sumit Kane: Conceptualization, Methodology, Fieldwork, Analysis, Writing – original draft. Madhura Joshi: Fieldwork, Data curation, Analysis, Writing- Reviewing. Sapna Desai: Writing- Reviewing. Ajay Mahal: Writing- Reviewing. Barbara McPake: Conceptualization, Writing- Reviewing and Editing.

## Data Availability

The authors do not have permission to share data.
